# 4,4′-(Propane-1,3-di­yl)dipiperidinium sulfate monohydrate

**DOI:** 10.1107/S1600536808025300

**Published:** 2008-08-16

**Authors:** E Yang, Xu-Chun Song, Rong-Qiang Zhuang

**Affiliations:** a College of Chemistry and Materials Science, Fujian Normal University, Fuzhou 350007, People’s Republic of China; b College of Chemistry and Chemical Engineering, Xiamen University, Xiamen 361005, People’s Republic of China

## Abstract

In the title compound, C_13_H_28_N_2_^2+^·SO_4_^2−^·H_2_O, extensive hydrogen-bonding inter­actions between the protonated 4,4′-(propane-1,3-di­yl)dipiperidinium ions, the sulfate anions and the water mol­ecules lead to a three-dimensional pillared and layered structure with the 4,4′-(propane-1,3-di­yl)­dipiperidinium ions acting as the pillars.
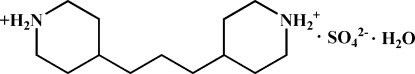

## Experimental

### 

#### Crystal data

C_13_H_28_N_2_^2+^·SO_4_^2−^·H_2_O
*M* *_r_* = 326.45
Monoclinic, 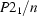

*a* = 6.2019 (2) Å
*b* = 22.5110 (5) Å
*c* = 12.0052 (3) Å
β = 100.439 (2)°
*V* = 1648.32 (8) Å^3^
*Z* = 4
Mo *K*α radiation
μ = 0.22 mm^−1^
*T* = 293 (2) K
0.22 × 0.14 × 0.09 mm

#### Data collection

Siemens SMART 1K CCD area-detector diffractometer
Absorption correction: multi-scan (*SADABS*; Sheldrick, 1996 ▶) *T*_min_ = 0.927, *T*_max_ = 0.98
13022 measured reflections
2932 independent reflections
2011 reflections with *I* > 2σ(*I*)
*R*_int_ = 0.066

#### Refinement

*R*[*F* ^2^ > 2σ(*F* ^2^)] = 0.048
*wR*(*F* ^2^) = 0.131
*S* = 1.03
2932 reflections
202 parameters
H atoms treated by a mixture of independent and constrained refinement
Δρ_max_ = 0.34 e Å^−3^
Δρ_min_ = −0.40 e Å^−3^

### 

Data collection: *SMART* (Siemens, 1996 ▶); cell refinement: *SAINT* (Siemens, 1996 ▶); data reduction: *SAINT*; program(s) used to solve structure: *SHELXS97* (Sheldrick, 2008 ▶); program(s) used to refine structure: *SHELXL97* (Sheldrick, 2008 ▶); molecular graphics: *DIAMOND* (Bergerhoff *et al.*, 1999 ▶); software used to prepare material for publication: *SHELXTL* (Sheldrick, 2008 ▶).

## Supplementary Material

Crystal structure: contains datablocks I, global. DOI: 10.1107/S1600536808025300/fj2138sup1.cif

Structure factors: contains datablocks I. DOI: 10.1107/S1600536808025300/fj2138Isup2.hkl

Additional supplementary materials:  crystallographic information; 3D view; checkCIF report

## Figures and Tables

**Table 1:** Hydrogen-bond geometry (Å, °)

*D*—H⋯*A*	*D*—H	H⋯*A*	*D*⋯*A*	*D*—H⋯*A*
O1*W*—H1*WA*⋯O4^i^	0.85	1.98	2.819 (3)	168
O1*W*—H1*WB*⋯O3^ii^	0.85	1.97	2.799 (3)	165
N1—H1*NA*⋯O4^iii^	0.94 (3)	2.09 (3)	2.904 (3)	144 (3)
N1—H1*NB*⋯O3^iv^	0.85 (3)	1.91 (3)	2.711 (3)	157 (3)
N2—H2*NA*⋯O1^v^	0.88 (3)	1.83 (3)	2.704 (3)	177 (3)
N2—H2*NB*⋯O4^vi^	0.92 (3)	2.02 (3)	2.845 (4)	149 (3)

Symmetry codes: (i) 
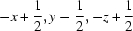
; (ii) 
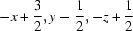
; (iii) 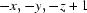; (iv) 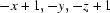; (v) 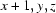; (vi) 
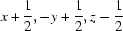
.

**Fig. 1.**
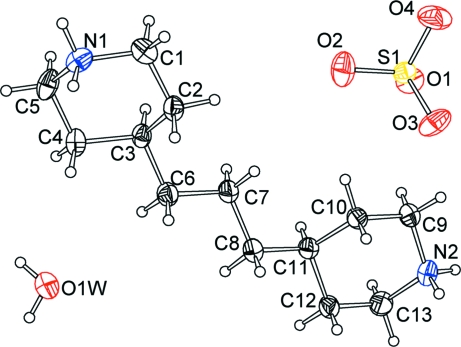
A view of the title compound, showing 30% probability displacement ellipsoids.

**Fig. 2.**
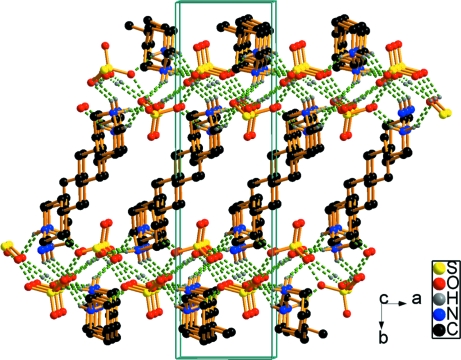
The three-dimensional structure of the title compound, showing the hydrogen bonding interactions (dashed lines).

## supplementary crystallographic information

### Comment

The asymmetric unit of the title compound, (I), consists of one protonated 4,4'-(propane-1,3-diyl)dipiperidinium ion, one deprotonated sulfate anion and one water molecule (Figure 1). Both protonated N ends of the 4,4'-(propane-1,3-diyl)dipiperidinium ion form N—H···O hydrogen bonds with the sulfate anion, as well as the water molecules form O—H···O hydrogen bonds with the sulfate anion, which leads to the formation of two-dimensional hydrogen-bonding layer parallel to the *ac* plane (Table 1 & Figure 2). The resulting layers are further pillared by the 4,4'-(propane-1,3-diyl)dipiperidinium ions to complete the three-dimensional structure.

### Experimental

A solution of 4,4-trimethylenedipiperidine (1 mmol), sulfuric acid (1 mmol) and H_2_O (10 ml) was slowly evaporated at room temperature, giving colorless single crystals suitable for X-ray analysis.

### Refinement

The H atoms bonded to C and O atoms were placed at calculated positions, and refined with isotropic displacement parameters, using a riding model [C—H 0.93Å and *U*_iso_(H) = 1.2*U*_eq_(C); O—H 0.85Å and *U*_iso_(H) = 1.5*U*_eq_(C)]. The H atoms bonded to N atoms were refined freely.

### Crystal data

**Table:**

C_13_H_28_N_2_^2+^·S_1_O_4_^2–^·H_2_O	*F*_000_ = 712
*M**_r_* = 326.45	*D*_x_ = 1.315 Mg m^−^^3^
Monoclinic, *P*2_1_/*n*	Mo *K*α radiation λ = 0.71073 Å
Hall symbol: -P 2yn	Cell parameters from 99 reflections
*a* = 6.2019 (2) Å	θ = 2.0–25.1º
*b* = 22.5110 (5) Å	µ = 0.22 mm^−^^1^
*c* = 12.0052 (3) Å	*T* = 293 (2) K
β = 100.439 (2)º	Prism, colorless
*V* = 1648.32 (8) Å^3^	0.22 × 0.14 × 0.09 mm
*Z* = 4	

### Data collection

**Table:**

Siemens SMART 1K CCD area-detector diffractometer	2932 independent reflections
Radiation source: fine-focus sealed tube	2011 reflections with *I* > 2σ(*I*)
Monochromator: graphite	*R*_int_ = 0.066
*T* = 293(2) K	θ_max_ = 25.1º
φ and ω scans	θ_min_ = 2.0º
Absorption correction: multi-scan(SADABS; Sheldrick, 1996)	*h* = −7→7
*T*_min_ = 0.927, *T*_max_ = 0.98	*k* = −26→26
13022 measured reflections	*l* = −13→14

### Refinement

**Table:**

Refinement on *F*^2^	Secondary atom site location: difference Fourier map
Least-squares matrix: full	Hydrogen site location: inferred from neighbouring sites
*R*[*F*^2^ > 2σ(*F*^2^)] = 0.048	H atoms treated by a mixture of independent and constrained refinement
*wR*(*F*^2^) = 0.131	*w* = 1/[σ^2^(*F*_o_^2^) + (0.0635*P*)^2^ + 0.5599*P*] where *P* = (*F*_o_^2^ + 2*F*_c_^2^)/3
*S* = 1.03	(Δ/σ)_max_ < 0.001
2932 reflections	Δρ_max_ = 0.34 e Å^−^^3^
202 parameters	Δρ_min_ = −0.39 e Å^−^^3^
Primary atom site location: structure-invariant direct methods	Extinction correction: none

### Special details

**Table:**

Geometry. All e.s.d.'s (except the e.s.d. in the dihedral angle between two l.s. planes) are estimated using the full covariance matrix. The cell e.s.d.'s are taken into account individually in the estimation of e.s.d.'s in distances, angles and torsion angles; correlations between e.s.d.'s in cell parameters are only used when they are defined by crystal symmetry. An approximate (isotropic) treatment of cell e.s.d.'s is used for estimating e.s.d.'s involving l.s. planes.
Refinement. Refinement of *F*^2^ against ALL reflections. The weighted *R*-factor *wR* and goodness of fit *S* are based on *F*^2^, conventional *R*-factors *R* are based on *F*, with *F* set to zero for negative *F*^2^. The threshold expression of *F*^2^ > σ(*F*^2^) is used only for calculating *R*-factors(gt) *etc*. and is not relevant to the choice of reflections for refinement. *R*-factors based on *F*^2^ are statistically about twice as large as those based on *F*, and *R*- factors based on ALL data will be even larger.

### Fractional atomic coordinates and isotropic or equivalent isotropic displacement parameters (Å^2^)

**Table:**

	*x*	*y*	*z*	*U*_iso_*/*U*_eq_	
S1	0.28582 (11)	0.18642 (3)	0.35885 (6)	0.0255 (2)	
O1W	0.7186 (3)	−0.20465 (10)	0.15532 (19)	0.0458 (6)	
H1WA	0.6074	−0.2274	0.1421	0.069*	
H1WB	0.8181	−0.2279	0.1410	0.069*	
O1	0.2335 (3)	0.19986 (9)	0.23718 (16)	0.0346 (5)	
O2	0.2510 (4)	0.12374 (10)	0.38116 (19)	0.0489 (6)	
O3	0.5164 (3)	0.20349 (10)	0.40084 (18)	0.0431 (6)	
O4	0.1467 (3)	0.22203 (10)	0.42124 (19)	0.0493 (7)	
N1	0.1395 (4)	−0.13893 (12)	0.4911 (2)	0.0310 (6)	
H1NA	0.026 (5)	−0.1498 (14)	0.529 (3)	0.046*	
H1NB	0.258 (6)	−0.1500 (14)	0.533 (3)	0.046*	
N2	0.8705 (4)	0.19250 (11)	0.0746 (2)	0.0279 (6)	
H2NA	0.986 (5)	0.1962 (14)	0.128 (3)	0.042*	
H2NB	0.851 (5)	0.2258 (14)	0.029 (3)	0.042*	
C1	0.1335 (5)	−0.07379 (13)	0.4752 (3)	0.0328 (7)	
H1A	0.1482	−0.0545	0.5484	0.039*	
H1B	−0.0068	−0.0624	0.4306	0.039*	
C2	0.3159 (5)	−0.05304 (13)	0.4160 (2)	0.0279 (7)	
H2A	0.4555	−0.0589	0.4661	0.033*	
H2B	0.2987	−0.0108	0.4007	0.033*	
C3	0.3192 (4)	−0.08591 (12)	0.3044 (2)	0.0249 (6)	
H3A	0.1859	−0.0756	0.2507	0.030*	
C4	0.3183 (5)	−0.15306 (12)	0.3261 (2)	0.0300 (7)	
H4A	0.3080	−0.1739	0.2546	0.036*	
H4B	0.4554	−0.1643	0.3739	0.036*	
C5	0.1292 (5)	−0.17174 (14)	0.3825 (2)	0.0351 (8)	
H5A	−0.0087	−0.1634	0.3326	0.042*	
H5B	0.1369	−0.2141	0.3972	0.042*	
C6	0.5170 (5)	−0.06873 (12)	0.2525 (2)	0.0297 (7)	
H6A	0.5259	−0.0957	0.1905	0.036*	
H6B	0.6483	−0.0743	0.3092	0.036*	
C7	0.5158 (4)	−0.00503 (12)	0.2080 (3)	0.0296 (7)	
H7A	0.3918	−0.0001	0.1466	0.036*	
H7B	0.4964	0.0222	0.2681	0.036*	
C8	0.7255 (5)	0.01124 (13)	0.1655 (3)	0.0315 (7)	
H8A	0.8465	0.0112	0.2293	0.038*	
H8B	0.7549	−0.0191	0.1129	0.038*	
C9	0.6696 (5)	0.18163 (13)	0.1245 (3)	0.0298 (7)	
H9A	0.5413	0.1820	0.0647	0.036*	
H9B	0.6536	0.2132	0.1774	0.036*	
C10	0.6836 (4)	0.12266 (12)	0.1851 (2)	0.0269 (7)	
H10A	0.5494	0.1160	0.2141	0.032*	
H10B	0.8042	0.1236	0.2491	0.032*	
C11	0.7181 (4)	0.07147 (12)	0.1069 (2)	0.0264 (7)	
H11A	0.5943	0.0712	0.0433	0.032*	
C12	0.9259 (5)	0.08438 (13)	0.0598 (3)	0.0332 (7)	
H12A	1.0508	0.0844	0.1215	0.040*	
H12B	0.9479	0.0530	0.0076	0.040*	
C13	0.9149 (5)	0.14331 (13)	−0.0008 (3)	0.0346 (8)	
H13A	1.0528	0.1506	−0.0258	0.042*	
H13B	0.7997	0.1420	−0.0673	0.042*	

### Atomic displacement parameters (Å^2^)

**Table:**

	*U*^11^	*U*^22^	*U*^33^	*U*^12^	*U*^13^	*U*^23^
S1	0.0231 (4)	0.0290 (4)	0.0237 (4)	−0.0011 (3)	0.0024 (3)	−0.0009 (3)
O1W	0.0439 (13)	0.0327 (13)	0.0623 (16)	0.0011 (10)	0.0131 (12)	−0.0080 (11)
O1	0.0342 (11)	0.0457 (14)	0.0209 (12)	0.0003 (9)	−0.0027 (9)	0.0051 (9)
O2	0.0630 (16)	0.0310 (14)	0.0508 (16)	−0.0044 (11)	0.0049 (12)	0.0120 (11)
O3	0.0239 (11)	0.0606 (15)	0.0401 (14)	−0.0115 (10)	−0.0070 (10)	0.0122 (11)
O4	0.0416 (13)	0.0637 (17)	0.0469 (15)	0.0067 (12)	0.0196 (11)	−0.0167 (12)
N1	0.0267 (13)	0.0376 (16)	0.0275 (16)	−0.0033 (11)	0.0019 (12)	0.0095 (12)
N2	0.0327 (14)	0.0249 (15)	0.0230 (14)	−0.0041 (11)	−0.0036 (11)	0.0046 (11)
C1	0.0350 (16)	0.0349 (19)	0.0274 (17)	0.0063 (14)	0.0028 (13)	−0.0008 (14)
C2	0.0298 (15)	0.0218 (16)	0.0298 (17)	−0.0013 (12)	−0.0008 (13)	−0.0008 (13)
C3	0.0267 (14)	0.0203 (15)	0.0258 (16)	−0.0005 (12)	−0.0008 (12)	0.0047 (12)
C4	0.0401 (17)	0.0219 (17)	0.0266 (17)	−0.0035 (13)	0.0022 (14)	−0.0004 (13)
C5	0.0396 (18)	0.0320 (18)	0.0291 (18)	−0.0142 (14)	−0.0058 (14)	0.0019 (14)
C6	0.0318 (16)	0.0244 (17)	0.0326 (18)	0.0010 (12)	0.0047 (13)	0.0020 (13)
C7	0.0324 (15)	0.0227 (16)	0.0357 (18)	0.0010 (12)	0.0110 (13)	0.0020 (13)
C8	0.0347 (16)	0.0229 (17)	0.0379 (19)	0.0002 (13)	0.0094 (14)	−0.0012 (13)
C9	0.0325 (16)	0.0251 (17)	0.0317 (18)	0.0027 (12)	0.0051 (13)	0.0021 (13)
C10	0.0300 (15)	0.0235 (17)	0.0286 (17)	0.0016 (12)	0.0093 (13)	0.0005 (13)
C11	0.0272 (15)	0.0248 (17)	0.0275 (17)	0.0003 (12)	0.0058 (13)	0.0011 (12)
C12	0.0407 (17)	0.0243 (17)	0.0398 (19)	−0.0020 (13)	0.0206 (15)	−0.0047 (14)
C13	0.0391 (17)	0.0342 (19)	0.0324 (19)	−0.0038 (14)	0.0114 (15)	−0.0033 (14)

### Geometric parameters (Å, °)

**Table:**

S1—O2	1.459 (2)	C4—H4B	0.9700
S1—O1	1.469 (2)	C5—H5A	0.9700
S1—O4	1.477 (2)	C5—H5B	0.9700
S1—O3	1.478 (2)	C6—C7	1.530 (4)
O1W—H1WA	0.8501	C6—H6A	0.9700
O1W—H1WB	0.8500	C6—H6B	0.9700
N1—C1	1.478 (4)	C7—C8	1.525 (4)
N1—C5	1.489 (4)	C7—H7A	0.9700
N1—H1NA	0.94 (3)	C7—H7B	0.9700
N1—H1NB	0.85 (3)	C8—C11	1.524 (4)
N2—C13	1.488 (4)	C8—H8A	0.9700
N2—C9	1.497 (4)	C8—H8B	0.9700
N2—H2NA	0.88 (3)	C9—C10	1.509 (4)
N2—H2NB	0.92 (3)	C9—H9A	0.9700
C1—C2	1.515 (4)	C9—H9B	0.9700
C1—H1A	0.9700	C10—C11	1.526 (4)
C1—H1B	0.9700	C10—H10A	0.9700
C2—C3	1.534 (4)	C10—H10B	0.9700
C2—H2A	0.9700	C11—C12	1.527 (4)
C2—H2B	0.9700	C11—H11A	0.9800
C3—C6	1.523 (4)	C12—C13	1.509 (4)
C3—C4	1.534 (4)	C12—H12A	0.9700
C3—H3A	0.9800	C12—H12B	0.9700
C4—C5	1.516 (4)	C13—H13A	0.9700
C4—H4A	0.9700	C13—H13B	0.9700
			
O2—S1—O1	111.60 (13)	C3—C6—C7	115.3 (2)
O2—S1—O4	108.18 (14)	C3—C6—H6A	108.4
O1—S1—O4	110.38 (13)	C7—C6—H6A	108.4
O2—S1—O3	110.79 (13)	C3—C6—H6B	108.4
O1—S1—O3	108.13 (12)	C7—C6—H6B	108.4
O4—S1—O3	107.69 (14)	H6A—C6—H6B	107.5
H1WA—O1W—H1WB	100.7	C8—C7—C6	113.0 (2)
C1—N1—C5	112.5 (2)	C8—C7—H7A	109.0
C1—N1—H1NA	108.6 (19)	C6—C7—H7A	109.0
C5—N1—H1NA	112.1 (19)	C8—C7—H7B	109.0
C1—N1—H1NB	111 (2)	C6—C7—H7B	109.0
C5—N1—H1NB	106 (2)	H7A—C7—H7B	107.8
H1NA—N1—H1NB	107 (3)	C11—C8—C7	114.3 (2)
C13—N2—C9	112.4 (2)	C11—C8—H8A	108.7
C13—N2—H2NA	108 (2)	C7—C8—H8A	108.7
C9—N2—H2NA	110 (2)	C11—C8—H8B	108.7
C13—N2—H2NB	105.2 (19)	C7—C8—H8B	108.7
C9—N2—H2NB	109.9 (19)	H8A—C8—H8B	107.6
H2NA—N2—H2NB	111 (3)	N2—C9—C10	110.9 (2)
N1—C1—C2	111.3 (2)	N2—C9—H9A	109.4
N1—C1—H1A	109.4	C10—C9—H9A	109.4
C2—C1—H1A	109.4	N2—C9—H9B	109.4
N1—C1—H1B	109.4	C10—C9—H9B	109.4
C2—C1—H1B	109.4	H9A—C9—H9B	108.0
H1A—C1—H1B	108.0	C9—C10—C11	111.7 (2)
C1—C2—C3	113.0 (2)	C9—C10—H10A	109.3
C1—C2—H2A	109.0	C11—C10—H10A	109.3
C3—C2—H2A	109.0	C9—C10—H10B	109.3
C1—C2—H2B	109.0	C11—C10—H10B	109.3
C3—C2—H2B	109.0	H10A—C10—H10B	107.9
H2A—C2—H2B	107.8	C8—C11—C10	112.6 (2)
C6—C3—C2	112.0 (2)	C8—C11—C12	112.5 (2)
C6—C3—C4	110.4 (2)	C10—C11—C12	107.8 (2)
C2—C3—C4	109.0 (2)	C8—C11—H11A	107.9
C6—C3—H3A	108.5	C10—C11—H11A	107.9
C2—C3—H3A	108.5	C12—C11—H11A	107.9
C4—C3—H3A	108.5	C13—C12—C11	112.3 (2)
C5—C4—C3	112.1 (2)	C13—C12—H12A	109.2
C5—C4—H4A	109.2	C11—C12—H12A	109.2
C3—C4—H4A	109.2	C13—C12—H12B	109.2
C5—C4—H4B	109.2	C11—C12—H12B	109.2
C3—C4—H4B	109.2	H12A—C12—H12B	107.9
H4A—C4—H4B	107.9	N2—C13—C12	111.0 (2)
N1—C5—C4	109.9 (2)	N2—C13—H13A	109.4
N1—C5—H5A	109.7	C12—C13—H13A	109.4
C4—C5—H5A	109.7	N2—C13—H13B	109.4
N1—C5—H5B	109.7	C12—C13—H13B	109.4
C4—C5—H5B	109.7	H13A—C13—H13B	108.0
H5A—C5—H5B	108.2		

### Hydrogen-bond geometry (Å, °)

**Table:**

*D*—H···*A*	*D*—H	H···*A*	*D*···*A*	*D*—H···*A*
O1W—H1WA···O4^i^	0.85	1.98	2.819 (3)	168
O1W—H1WB···O3^ii^	0.85	1.97	2.799 (3)	165
N1—H1NA···O4^iii^	0.94 (3)	2.09 (3)	2.904 (3)	144 (3)
N1—H1NB···O3^iv^	0.85 (3)	1.91 (3)	2.711 (3)	157 (3)
N2—H2NA···O1^v^	0.88 (3)	1.83 (3)	2.704 (3)	177 (3)
N2—H2NB···O4^vi^	0.92 (3)	2.02 (3)	2.845 (4)	149 (3)

Symmetry codes: (i) −*x*+1/2, *y*−1/2, −*z*+1/2; (ii) −*x*+3/2, *y*−1/2, −*z*+1/2; (iii) −*x*, −*y*, −*z*+1; (iv) −*x*+1, −*y*, −*z*+1; (v) *x*+1, *y*, *z*; (vi) *x*+1/2, −*y*+1/2, *z*−1/2.
